# “Silymarin”, a Promising Pharmacological Agent for Treatment of Diseases

**Published:** 2011

**Authors:** Gholamreza Karimi, Maryam Vahabzadeh, Parisa Lari, Marziyeh Rashedinia, Mohammad Moshiri

**Affiliations:** 1 *Medical Toxicology Research Center, School of Pharmacy, Mashhad University of Medical Sciences, Mashhad, Iran *; 2 *Department of Pharmacodynamy and Toxicology, School of Pharmacy, Mashhad University of Medical Sciences, Mashhad, Iran*

**Keywords:** Antioxidant, Cancer, Liver, Medicinal plant, Silymarin

## Abstract

Widespread use of herbal drugs because of their protective effects on different organs toxicity has been shown in many studies. These protective effects have been illustrated in the fields of nephrotoxicity, hepatotoxicity, viral hepatitis, cancer, *in vitro* fertilization, neurotoxicity, depression, lung diseases, prostate diseases etc. Silymarin has cytoprotection activities due to its antioxidant activity and radical scavenging. The possible known mechanisms of action of silymarin protection are blockade and adjustment of cell transporters, p-glycoprotein, estrogenic and nuclear receptors. Moreover, silymarin anti-inflammatory effects through reduction of TNF-α, protective effects on erythrocyte lysis and cisplatin-induced acute nephrotoxicity have been indicated in some studies. Silymarin has also inhibited apoptosis and follicular development in patients undergoing IVF. Basis on such data, silymarin can be served as a novel medication in complementary medicine.

## Introduction

*Silybum marianum* L. (Milk thistle), a member of Carduus marianum family, is an ancient medicinal plant which has been used for centuries for treatment of different diseases such as liver and gallbladder disorders, protecting liver against snake bite and insect stings, mushroom poisoning and alcohol abuse (1). This plant can be found in Kashmir, North America, Canada and Mexico with large leaves and a reddish-purple flower that are all thorny and the medicinal part of the plant is either the seeds or fruits (2).

Milk thistle was first grown in Europe and used as a liver tonic as it was said to be able to open the obstructions of the liver and spleen, and thereby was good for jaundice (Nicolas Culpepper, 1616-1654)(3). Moreover, this herb has been used for centuries as a natural treatment for upper gastrointestinal tract and digestive problems, liver and biliary tract diseases, menstrual disorders and varicose veins (4,5). 

**Figure 1.**
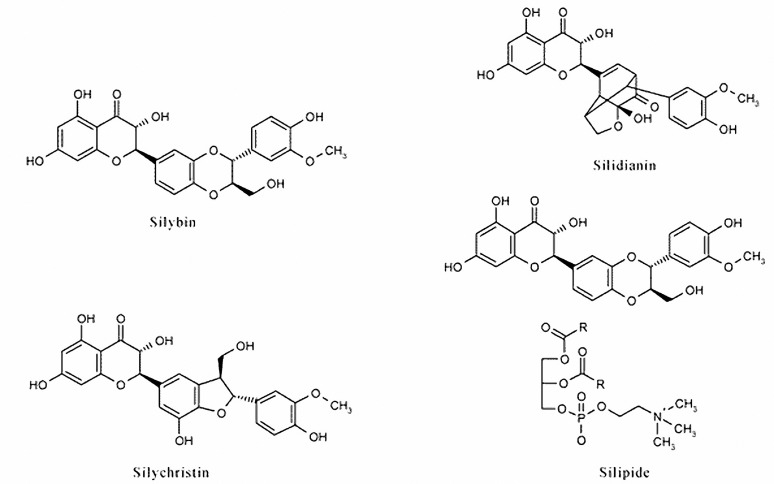
Chemical structure of some of the silymarin components.

The very first usage of Milk thistle, however, was for its hepatoprotectant and antioxidant activities. Silymarin is the active component of this herb, which is a complex of other components, mainly silybin A, silybin B, isosilybin A, isosilybin B and also other flavonolignants such as silychristin, neosilyhermin, silyhermin and silydianin which exists in its fruit and seeds more than the other parts (Figure 1) (2, 6-8).

Silymarin effects have also been indicated in various illnesses of different organs such as prostate, lungs, CNS, kidneys, pancreas, and skin (9). 

Silymarin has besides antifibrotic, immunomodulating, anti-inflammatory effects as well as antioxidant properties by scavenging free radicals and increasing the glutathione concentrations, so that it can be used in hepatitis and hepatic cirrhosis treatment and in mushroom poisoning (5, 7,10).

According to pharmacological studies, silymarin has been accepted as a safe herbal product, since using the physiological doses of silymarin is not toxic unless the improper administration of therapeutic dosages (10-12).

The main adverse effects reported are headaches, gastroenteritis and dermatological symptoms, among them gastrointestinal symptoms are the most common (1).

Milk thistle extract is now marketing as silymarin and silybinin capsules and tablets with an improved bioavailability under the trade names like Livergol, Silipide and Legalon (6). 

In animal models, silymarin active components had protective effects against hepatotoxic medications for chemotherapy of tuberculosis (13).

Antioxidant properties have been reported for silymarin, which increases the superoxide dismutase activity within the erythrocytes and lymphocytes (3). Silymarin can also alleviate hepatocyte membrane and thus prevent the xenobiotics from going into the cell via enterohepatic circulation. Silymarin can slightly bind to the iron and inhibit human hepatocyte glutathione reduction (3). Silymarin is able to modulate the immune system, and enhances the IFN-γ, IL-4 and IL-10 secretion in cultures containing lymphocytes. Its anti-neoplastic effects are related to the pro-angiogenic factors and growth inhibition, induction of endothelial cells apoptosis through a p53-dependent pathway involving Bcl-2/Bax, cytochrome C release, Apaf-1, and activation of caspase-3 and PARP (3,14).

**Figure 2.**
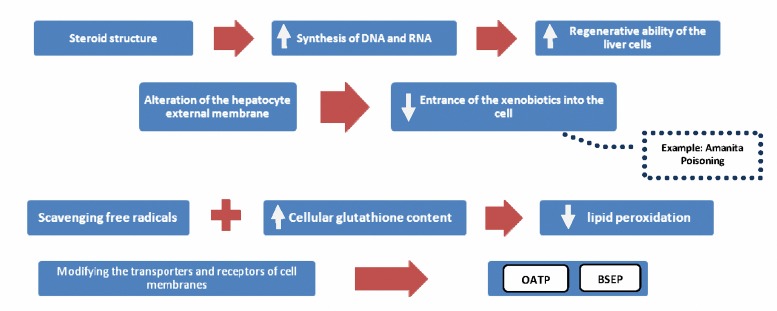
Different mechanisms of action of silymarin are as follow: Increasing the regenerative ability of the liver cells by enhancing the synthesis of DNA and RNA, as silymarin has a steroid structure; Altering the structure of the hepatocyte external membrane, that prevents entrance of the xenobiotics into the cell (poisoning with Amanita mushroom is a noteworthy example of such mechanism); Scavenging free radicals and increasing the cellular content of glutathione that leads to the inhibition of lipid peroxidation; Another mechanism of action of silymarin is modifying the transporters and receptors of cell membranes such as ABC transporters (P-gp), organic anion uptake transporter peptides (OATP), bile salt export pump, and TNF-α-dependent transporters (2,4).

## Mechanism of action

Silymarin acts through the various mechanisms as follow (2,4): It undergoes enterohepatic circulation and shifts from plasma to bile, which finally concentrates in hepatocytes (Figure 2).

**Applications**

***Hepatoprotection***

Liver is the key organ of metabolism and excretion is continuously and variedly exposed to xenobiotics because of its strategic placement in the body. Toxins absorb from the intestinal tract first enter the liver resulting in a variety of liver disorders. Thus, liver diseases remain one of the serious health problems. Liver damage ranges from acute hepatitis to hepatocellular carcinoma, being caused through apoptosis, necrosis, inflammation, immune response, fibrosis, ischemia, altered gene expression, and regeneration (15). 

For many years, silymarin has been used as a “hepatoprotectant”. Although the mechanism of action is not completely demonstrated, silymarin has been reported to have antioxidant, immunomodulatory, antifibrotic, antiproliferative, and antiviral properties. Silymarin has a short half-life and quick conjugation in the liver and principal excretion in bile. In means of controlling hepatic inflammation *in vivo*, it should be used with high or repeated oral doses (16).

As mentioned previously, silymarin hepatoprotective properties are free radical scavenging and raising the cellular content of glutathione that lead to the lipid peroxidation inhibition, increasing membrane stability in exposure to xenobiotics, steroid-like effect via adjustment of nuclear expression and reducing the deposition of collagen fibres as silymarin inhibits the conversion of stellate hepatocytes into myofibroblasts. In addition, silymarin/silybinin increases ribosomal protein synthesis by means of stimulating RNA polymerase I (4). 

Silybinin inhibits elevated intra-hepatic messenger RNA (mRNA) levels of IL-2, IL-4, IFN-γ, and TNF-α significantly. It also reduces the alanine aminotransferase and aspartate aminotransferase levels and suppressed the apoptosis in hepatocytes (4,16).

*In vitro* experiments have been verified that two components of silymarin, silybin A and B are able to inhibit T-cell proliferation and proinflammatory cytokine secretion in a dose-dependent manner. High oral doses of silymarin in human have controlled hepatic inflammation in chronic liver disease (16).

In non-alcoholic fatty liver disease (NAFLD), elevation of circulating free fatty acids and their levels correlate with disease severity. Agents that prevent or decrease hepatocyte death due to free fatty acids can be a potential medication for NAFLD (17). 

In animal experiments, silymarin and silybinin are indicated to have protective effects on rat or mouse liver against hepatotoxicity in acute ethanol intoxication, carbon tetrachloride, cisplatin, thioacetamide, thallium, D-galactosamine and acetaminophen (10).

Treatment with ethanolic extract (100 mg/kg bw) of silymarin seed most significantly declined the rats liver enzymes while using against carbon tetrachloride-induced (2 ml/kg bw) liver damage. Moreover, in oxidative experiment, ethyl acetate extract of silymarin showed the most enhancements in glutathione level and HDL/LDL (4,18).

Pre-treatment of male mice with silymarin modulated the alteration of oxidative stress, cell cycle, cytoskeletal network, cell–cell adhesion, extra-cellular matrix, inflammation, apoptosis, cell-signaling and intermediary metabolism that was induced by pyrogallol. These effects leaded to the differential expression of 79 genes/transcripts (27 up-regulated and 52 down-regulated) in comparison to the pyrogallol treated group. The results showed that, effects of silymarin could be due to its multiple functions as well as its antioxidant activity (19).

Findings in animal and human studies revealed the highest concentrations and therefore the more effects of silymarin in the liver (16).

Fibrosis development is the major outcome of chronic liver infections that commonly occurs in immunecompetents (16). Polyak *et al* (2007) showed that silymarin inhibits the replication of an infectious HCV genotype 2a strain (JFH1) in hepatoma cell culture (20). These effects are the result of silymarin compounds being able to inhibit HCV RNA-dependent RNA polymerase activity (21). Two phase II trials are being performed by the National Center for Complementary and Alternative Medicine (NCCAM) which investigates using silymarin as a treatment in hepatitis C (3).

In another study, silymarin decreased amiodarone levels and amiodarone-induced lysosomal phospholipidosis in the liver (4). 

A primary role for MRP2, a cell membrane transporter, has been showed in the biliary excretion of silymarin conjugates. Chronic liver disease can manage to change the hepatic expression of MRP2. Primarily silydianin is glucuronidated and then excreted into bile suggesting that it might be used as a specific probe for MRP2 substrate (22).

In mouse model of alcoholic liver diseases that oxidative stress and inflammation were the main causes of the pathogenesis, silymarin was observed to pose hepatoprotective effects by producing the tumor necrosis factor (TNF) and decreasing the serum alanine aminotransferase (ALT) activity, which inhibits lipid peroxidation, and increases the intracellular GSH content (6). 

Oral post-treatment with silymarin (50 mg/kg for 30 days) in rats extensively inverted the liver tissue changes induced by diethylnitrosamine and presented a relatively full protection (23). 

HepG2 -cells death occurs via inhibition of Akt kinase stimulated by palmitate exposure and silymarin prevents this inhibition as it has hepatoprotective activity different from its antioxidant property (17).

In a clinical trial using silymarin in alcoholic patients with confirmed liver cirrhosis, silymarin (150 mg/three times per day) administered for two years and no influence of silymarin was seen in case of survival and clinical course of the disease in comparison to the sham group (6).

Currently, silymarin is mainly used as a remedy for *Amanita phalloides *(death cup fungus) intoxication where silymarin plays a role in hepatoprotection through the mechanisms such as stopping the α-amanitin entero-hepatic cycle, prevention of phalloidin and α-amanitin binding to membranes of hepatocyte, and antagonising the α-amanitin membrane transporting (18,24). 

In a retrospective clinical report of 205 patients with amanita poisoning, no fatality observed after administration of intravenous silybinin (20–50 mg/kg/body weight-daily) to 16 individuals (24). As the results have been conflicting, clinical efficacy of silymarin in chronic liver diseases has not yet been demonstrated (25-26). 

***Prevention and treatment of Cancers***

Effects of silymarin or silybinin on breast cancer (27-28), ovarian cancer, lung cancer (29), skin cancer (30), prostate cancer (31-33), cervical cancer, bladder cancer, liver carcinoma (34), and colon cancer (35), have been reported (6).

Mechanism of cytoprotective activity of silybin related to antioxidative and radical-scavenging effects as well as the specific receptor interaction and modulation of a variety of cell-signaling pathways e.g. NF-kappa B, suppression of EGFR-MAPK/ERK1/2 signaling and IGF-receptor signaling (9). In addition, Anti-apoptotic effect of silymarin against UV irradiation has been revealed by up-regulation of tumor-suppressor genes p53- and p21CIP1 (4,36).

Silymarin has been shown to have anti-angiogenic property in different kinds of cancers, which is one of the basic treatments of cancer. Moreover, previous studies have shown silymarin and silybin anti-angiogenic activity in human umbilical vein endothelial cells (HUVEC) dose-dependently by mechanism of decreasing of vascular endothelial growth factor (VEGF) and matrix metalloproteinase-2 (MMP-2) secretion (1,6). 

Down-regulation of EGFR signaling by silymarin and silibinin occurs via various mechanisms such as the inhibition of growth factors expression and secretion, preventing growth factor binding and activation of EGFR and destruction of mitogenic procedures causing anti-cancer effectiveness in tumor cells (37). 

This inhibition of mitogenic signaling pathways in prostate carcinoma leads to alteration of cell cycle regulators, inhibition of growth and androgen-independent prostate carcinoma cells loss and expression of insulin-like growth factor-binding protein 3 (1).

However, numerous *in vitro* and *in vivo* experiments containing cancer models did not show significant dissimilarity in biological activity between silymarin and silybin (11).

Malondialdehyde results from lipid peroxidation and leads to MDA-DNA adduct formation, which causes frame shift mutations as an association between oxidative stress and human cancers (38). 

Treatment with silymarin considerably reduces the generation of MDA-DNA adducts and hepatocellular carcinoma serum markers such as alpha-fetoprotein, carcinoembryonic antigen, aminotransferase, alkaline phosphatase, lactate dehydrogenase, gamma-glutamyltransferase and 5´-nucleotidase (38).

Multidrug resistance is one of the main problems of successful cancer treatment, which is related to P-glycoprotein (P-gp) or multidrug resistance-associated protein 1 (MRP1) over expression. Silymarin elevates absorption and bioavailability of chemo-pharmaceutics such as daunomycin, vinblastine, and doxorubicin in cancerous cells by inhibition of P-glycoprotein (P-gp), MRP1-mediated drug carrier and breast cancer resistance protein (BRCP) (4, 6,9).

Silymarin can be applied as a co-treatment with the other chemotherapeutics agents while silybin is mainly useful as a hepatoprotective substance against chemotherapeutics-induced oxidative stress. Silybinin growth inhibitory effects and apoptotic efficacy have been also illustrated in prostate carcinoma cell culture and rat prostate cancer cells (33).

Moreover, silymarin inhibits β-catenin increase, which will suppress the proliferation of hepatocellular carcinoma HepG2 cells. β-catenin is a vital factor in cell adhesion complex. It stimulates T-cell transcription factor and plays an important role in regulation of oncogenic process, as well as anti-apoptotic effects in various cancers. On the other hand, mitochondrial membrane potential of HepG2 cells decreases by silymarin that causes disruption of membrane permeability so that cytochrome C transfers from the intermembrane space to the cytoplasm (11). 

While apoptosis is induced by p53 through activating pro-apoptotic genes, levels of p53 increase by silymarin treatment in a dose dependently manner which leads to cytochrome C release, activating many pro-apoptotic genes such as APAF-1 and caspase-9. Hence, it has been demonstrated that silymarin has the growth inhibitory effect by cell proliferation suppression and apoptosis induction (11). 

***Renal protection***

The effect of silymarin has been tested in alloxan-induced diabetes mellitus models in rats. Alloxan produces reactive oxygen species (H_2_O_2_, •O_2_ and •OH) (39), which injure renal tissue (40-41). Silymarin was administrated 20 days after 9 weeks treatment with alloxan and it was effective on the renal tissue injuries. It has antioxidant effects via increase of gene expression of antioxidant enzymes and a number of the most important protection mechanisms against free radicals damage containing super- oxide dismutase, glutathione peroxidase, and catalase. Therefore, silymarin can be used as a drug for diabetic nephropathy therapy (42). 

Oxidative stress (ROS) reduces glomerular filtration. Treatment with silymarin or vitamin E improved alteration in serum creatinine concentrations in the gentamicin-treated dogs (43). 

In another study, cisplatin and ifosfamide-induced renal toxicity can be antagonized by silymarin without reducing anti-tumor efficacy of these drugs (6, 44-45).

Ferric nitrilotriacetate (Fe-NTA) induced nephrotoxicity and cancer of kidney by causing redox active iron-made reactive oxygen species and lipid peroxidation (LPO) that can damage cell membrane and molecules such as DNA. The formation of 8-hydroxy guanosine leads to mutation in DNA (46). 

Silymarin has supportive effects on Fe-NTA induced LPO. This protection can be related to its antioxidant and free radical scavenging actions. NFκB (nuclear factor kappa B) causes activation of numerous oncogenic process, for instance cellular inflammation, proliferation, inhibition of apoptosis via enhancing of the expression of downstream genes (nitric oxide synthase, cyclooxygenase 2 and proinflammatory cytokines for example tumor necrosis factor alpha (TNF-α) and interleukin-6). Thus, suppression of NFκB is known as a helpful plan to control the carcinogenic effects. NFκB activation can be suppressed by silymarin because of some stimulant like phorbol ester, lipopolysaccharide, okadaic acid and ceramide. These results proposed silymarin as a strategy for renal carcinogenesis treatment because of decreasing some tumor inducer factors in animal models (46). 

In a human study administration of silymarin (210 mg/day) for 8 weeks in peritoneal dialysis patients inhibited the effects of pro-inflammatory cytokines especially TNF- (47).

Inhibitory effect of TNF-, on erythropoiesis and suppression of bone marrow via prevention of producing the erythroid colony forming units (E-CFU), an early development precursor of red cells, causes problems in hematological status in advanced renal failure patients. In this study, 40% of the patients revealed a significant response and hemoglobin concentrations were increased after 8 weeks of silymarin administration. As a result, silymarin can be supposed in treating inflammatory anemia in peritoneal dialysis patients (47).

***Neuronal effect***

High oxygen utilization, huge amounts of polyunsaturated fatty acids, elevated levels of free iron ions and low antioxidants defenses all together make the brain tissue vulnerable to reactive oxygen species injuries (48). Silymarin when administered at a dose of 200 mg/kg/day, strongly reduced the proteins oxidation in hippocampus and cortex of elderly rats in comparison to the young ones. 

Silymarin can be used as a choice compound against Alzheimer disease in which the protein oxidation is an important early occasion. According to previous studies, silymarin has antioxidant activities in the central nervous system, which enables it to enter the CNS via the blood–brain barrier (BBB) (48-51). 

Administration of 200 mg/kg silymarin also reduced the rotational behavior caused by 6-hydroxydopamine (6-OHDA) in hemi-parkinsonian rats and the substantia nigra pars compacta neurons were protected against its toxicity, suggesting a dose-dependently neuroprotection effect of silymarin against 6-OHDA toxicity, through oxidative stress decline and by means of an estrogenic pathway (52).

Silymarin have also known to be able to elevate some neurotransmitters concentration in brain. A study on modified forced swimming test in mice used aqueous and ethanolic extracts of silymarin. Results showed that ethanolic extract had no effect on the duration of mice immobility while the aqueous extract significantly diminished it, concluding that aqueous extract of silymarin has antidepressant effect in animal models (53).

***Immunomodulation***

Based on a splenocytes examination by flow cytometric method, silymarin significantly reduced number of CD3+ T-lymphocytes and the CD4+ population with 10 mg/kg dose. In this study, mice were exposed to different doses of silymarin (0, 10, 50 or 250 mg/kg, intraperitoneally, once a day for 5 days). In the lowest dose group there was an increase in proliferation of phytohemagglutinin-induced T-lymphocyte. Doses of 10 and 50 mg/kg of silymarin increased B-lymphocyte blastogenesis induced by LPS (lipopolysaccharide) and reduced the expression of IL-2 and IL-4. However, it increased expression of TNF-α, iNOS, IL-1β and IL-6 mRNA dose-dependently. As a result, ‘*in vivo*’ exposure to low doses of silymarin suppresses function of T-lymphocyte and stimulates the inflammatory pathways at higher doses (4). 

In further studies, silymarin significantly decreased IL-2 and interferon gamma (IFN-γ) production and blocked nuclear translocation of transcription factor κB (NF-κB) which activates IL-2 transcription. It can be concluded that silymarin suppresses activation and proliferation of T cells, particularly by affecting pathways of NF-κB activation or translocation (54).

***Protective effect on pancreas***

Silymarin can increase serum insulin, reduce serum glucose and rise of antioxidant enzymes and glutathione. as well as recover endocrine function and pancreatic morphology in diabetic models (42). 

In addition, silybin has chemoprotectant effect and can improve pancreatic function after exposure to toxic agents leading to damages (1, 41, 55-56).

Alloxan is a substance, which provokes diabetes mellitus by necrosing beta pancreatic cells and production of free radicals. Concurrent treatment with alloxan and silymarin in alloxan- induced diabetes mellitus rats prevented high plasma glucose levels and damages in pancreatic cells within 3 days of the first dose of silymarin administration and 5 days later the mentioned changes were completely prevented. Resulting from these data, silymarin can be considered as a potential drug for diabetes treatment (1).

***Preventing effect against hemolysis***

Reactive oxygen species can damage the cell membrane structure and destruct protein functions, especially enzymes. Membrane of erythrocytes are sensitive to lipid peroxidation in patients with glucose-6-phosphate dehydrogenase deficiency, sickle cell anemia and β-thalasemia disease (57).

According to a study on model of chain oxidation of lipids and proteins induced red blood cells hemolysis by 2, 2’-azobis–(2-amidinopropane) (AAPH), a water- soluble radical generator, silymarin increased the lag time of hemolysis and stabilized the cell membrane by reducing the rate and the total content of glutathione loss in erythrocytes. It also decreased the concentration of peroxyl radicals derived from AAPH as a chain-breaking antioxidant and radical scavenger (57, 58).

***Antiosteoporotic and selective estrogen receptor modulator***

In one study silymarin intake could increase the parathormone concentration in ovariectomized -induced bone loss that had leaded to trabecula thickness of the femur and had a positive effect on bone formation. Estrogenic effects of silymarin lead to increasing the uterine weight and endometrial height, in addition to hypertrophy of luminal epithelium. However, silymarin had no estrogenic effects on the hypothalamo/pituitary axis (no effects on serum LH and FSH levels). Uncontrolled silymarin dose could elevate risk of endometrial hyperplasia (59).

***Protective effect against environmental toxin***

In a study involving healthy volunteers, the cytotoxic effect of Benzo(a) pyrene on peripheral blood mononuclear cells was prevented by silymarin through stabilizing cell membranes, increasing the GSH/GSSG ratio, restoration of glutathione metabolizing enzymes, elimination agents produced from lipid peroxidation and protein oxidation and functional stimulation of the antioxidant enzymes such as catalase and superoxide dismutase (60). 

**Dosage forms**

The available forms of Milk thistle are capsules, tablet, tincture and intravenous solution. Adult dosage in terms of hepatoprotection is 420 mg/day of extract (standardized to 70-80% silymarin) three times a day for 6-8 weeks. Maintenance dose is 280 mg/day. Intravenous solution is used for cyclopeptid mushroom poison in dose of 33 mg/kg/day for approximately 81.67 hr (5).

**Toxicology and adverse effects**

Silymarin acceptability is good and just a gentle gastrointestinal disturbance and mild allergic reactions, urticaria, nausea, headache, joint pain, itching, and mild laxative symptoms have been reported. In animal studies, silymarin has been reported to be nontoxic and symptom free with the maximum oral doses of 2500 and 5000 mg/kg. It has been also illustrated that silymarin is not teratogen and had no post-mortem toxicity (2, 5, 33).

As there was not significant toxicity of silymarin reported in human studies, this substance can be used with anti-tuberculosis drugs as a supplement added to the diet (13). Although silymarin is safe, little is known about its mechanism of action and drug/food interactions (3).

## Conclusion

Silymarin possess wide range of *in vitro* and *in vivo* mechanisms, such as antioxidant, anti-inflammatory, dose dependent anti-apoptotic and modifying cell transporters. Hence, it can be used as a promising medication in complementary medicine.
